# Effects of Short Periods of Incubation During Egg Storage (SPIDES) on Egg Quality, Hatching Performance, and Chick Quality in Long-Term-Stored Chukar Partridge Eggs

**DOI:** 10.3390/ani16182819

**Published:** 2026-09-08

**Authors:** Mahmut Şamil Şamlı, Kemal Kırıkçı

**Affiliations:** 1 Department of Animal Science, Faculty of Veterinary Medicine, Hatay Mustafa Kemal University, Hatay 31060, Türkiye; mahmutsamil.samli@mku.edu.tr; 2 Department of Animal Science, Faculty of Veterinary Medicine, Selçuk University, Konya 42130, Türkiye

**Keywords:** *Alectoris chukar*, chick quality, egg quality, long-term egg storage, SPIDES, hatchability

## Abstract

This study investigated the effects of short periods of incubation during egg storage (SPIDES) on egg quality, hatching performance, and chick quality in *Alectoris chukar* eggs subjected to extended storage durations. A total of 800 eggs collected from a 60-week-old chukar partridge breeder flock were allocated to eight experimental subgroups in a 4 × 2 factorial design, with storage period (35, 42, 49, and 56 days) and treatment (control or SPIDES) as the main factors (100 eggs/subgroup). All eggs were stored at 9–12 °C and 75% relative humidity in a pointed-end-up position with 24 rotations per day for their assigned storage duration (35, 42, 49, or 56 days). In the SPIDES groups, eggs were incubated for 2 h at 37.7 °C and 59% relative humidity in 7-day intervals, commencing on day 28 of storage. As storage duration increased, Haugh unit, albumen index, and yolk index decreased significantly (*p* < 0.001), while yolk ratio (*p* < 0.05) and yolk-to-albumen ratio (*p* < 0.01) increased. The overall hatchability decreased at 49 and 56 days of storage, while the hatchability of fertile eggs was lowest at 56 days (*p* < 0.05). Early embryonic mortality increased significantly at 56 days of storage (*p* < 0.05). SPIDES had a limited effect on most egg quality traits, including Haugh unit, albumen index, and egg component weights, but significantly improved the hatchability and hatchability rate of fertile eggs, reduced early embryonic mortality, and enhanced the overall chick quality score (*p* < 0.05). These findings indicate that chukar partridge eggs exhibit considerable resistance to long-term storage; however, SPIDES represents a beneficial management strategy for maintaining embryonic viability when storage duration exceeds practical limits.

## Simple Summary

In many poultry species, storage beyond 7–14 days can reduce egg quality, hatchability, and chick quality. This study examined chukar partridge eggs stored for 35, 42, 49, or 56 days, representing unusually prolonged storage conditions, and evaluated the effects of short periods of incubation during egg storage (SPIDES). Egg quality and hatching performance decreased as storage duration increased, with the negative effects being most evident in chukar partridge eggs after 49 and 56 days. SPIDES had little effect on most egg quality traits, such as Haugh unit, albumen index, and egg component weights, but improved hatchability and chick quality and reduced early embryo deaths at 56 days. These results demonstrate that chukar partridge eggs are resistant to long-term storage conditions and that SPIDES may help mitigate the negative effects of long-term storage in partridge eggs.

## 1. Introduction

The poultry industry is a major source of animal protein worldwide and has benefited from advances in genetics, nutrition, and environmental management [1,2]. Hatchery performance is an important component of poultry production because it affects the number and quality of chicks produced and may influence their subsequent health, welfare, and growth [3,4]. Egg storage is one of the main pre-incubation factors affecting embryonic viability and hatching performance [5].

In commercial hatcheries, logistical constraints routinely necessitate the storage of hatching eggs for several days to weeks before incubation. During this period, a series of physicochemical and biological changes occur within the egg that collectively compromise embryonic viability, hatching performance, and chick quality [6,7]. As storage duration extends beyond the species-specific tolerance threshold, carbon dioxide (CO_2_) loss through shell pores drives albumen pH upward, disrupting the ovomucin–lysozyme complex that maintains albumen viscosity; simultaneously, the vitelline membrane undergoes osmotic-driven weakening, allowing water to migrate from the albumen into the yolk [8]. These changes are reflected in declining Haugh unit values, reduced albumen and yolk indices, and progressive structural disorganization of the blastoderm [9].

The tolerance to long-term storage varies substantially among avian species. In commercial broiler breeders, hatchability begins to decline after 7 days of storage, with pronounced losses reported beyond 14 days [10]. Japanese quail eggs also show reductions in egg quality and hatchability as storage duration increases [11], while negative effects in pheasant eggs have been reported after storage periods of 8–14 days [12]. In comparison, partridge eggs appear to tolerate longer storage periods. Previous studies reported that chukar partridge eggs could be stored for up to 28 days without a marked reduction in hatching performance under controlled conditions [13,14,15,16]. Similar findings have been reported for rock partridge, red-legged partridge, and game quail eggs [14,17,18]. The biological basis of this apparent tolerance is not fully understood, but it may be related to the period of egg accumulation before natural incubation begins [19].

However, information on the egg quality and hatching performance of *A. chukar* eggs stored for longer than 28 days remains limited. It is also unclear whether management practices, such as supportive interventions during storage, can reduce the negative effects of such long-term storage in this species.

Short periods of incubation during egg storage (SPIDES) involves warming eggs briefly at incubation temperature one or more times during storage [20]. This practice resembles the intermittent warming of eggs in natural nests before continuous incubation begins. SPIDES may advance the embryo from the Eyal-Giladi and Kochav stage X to later developmental stages that are more tolerant of storage, reduce cell death, and help maintain blastoderm viability [21,22]. Studies in broiler breeder eggs have shown that SPIDES can improve hatchability and chick quality after long-term storage [21,23,24]. However, the response may depend on the species and the timing and duration of warming. In red-legged partridge eggs, a single pre-storage incubation treatment did not improve hatchability, and a longer treatment reduced hatchability after extended storage [25]. Therefore, warming protocols developed for chickens cannot be assumed to be equally suitable for partridge species.

Therefore, this study aimed to determine the effects of storage for 35, 42, 49, and 56 days on egg quality, hatching performance, embryonic mortality, and chick quality in chukar partridge eggs. This study also evaluated whether SPIDES, applied for 2 h at 37.7 °C every 7 days from day 28 of storage, could reduce the negative effects of prolonged storage. We hypothesized that prolonged storage would impair egg quality and hatching outcomes and that SPIDES would reduce these effects, particularly for the longest storage durations.

## 2. Materials and Methods

### 2.1. Ethics Statement

All procedures were conducted in accordance with national regulations on the use of animals in research. The study protocol was approved by the Ethics Committee of the Experimental Animals Production and Research Center, Faculty of Veterinary Medicine, Selçuk University (protocol code 2024/111; meeting No. 2024/07; approval date: 4 July 2024).

### 2.2. Animals, Egg Collection, and Selection

Experimental eggs were collected from a 60-week-old chukar partridge (*Alectoris chukar*) breeder flock maintained for breeding and hatching-egg production housed at the Bahri Dağdaş International Agricultural Research Institute (Konya, Türkiye). The birds were housed in 20 semi-open wire-mesh pens (6.0 × 1.2 × 1.5 m), each containing 30 females and 10 males. Natural photoperiod was supplemented with artificial lighting, increasing by 1 h per week from week 36 to achieve a final 16 h light/day schedule. Feed and water were provided ad libitum. The nutrient composition of the breeder diet is presented in Supplementary Table S1.

Eggs were collected twice daily (09:00 and 16:00 h) and subjected to visual inspection. Eggs with cracks, excessive soiling, or malformed shells were excluded. Egg weight was constrained to 18–24 g; eggs outside this range were excluded. Each egg was individually numbered, assigned a group code, and weighed using a precision balance (PFB 300-3; KERN & SOHN GmbH, Balingen, Germany) with an accuracy of ±0.01 g (W0).

### 2.3. Experimental Design

A 4 × 2 factorial design was used, with storage duration (35, 42, 49, and 56 days) and treatment (control or SPIDES) as fixed factors. Eight experimental subgroups were formed, each containing 100 eggs, for a total of 800 eggs. Each experimental subgroup consisted of 100 individually numbered eggs, and measurements were recorded on an individual egg basis. Eggs within each storage duration group were randomly allocated to control and SPIDES groups. To enable simultaneous incubation of all groups, eggs were introduced in 7-day intervals. The experimental work was conducted between July and October 2024 at the Research and Application Farm of Selçuk University Faculty of Veterinary Medicine (38°02′14″ N, 32°30′20″ E).

### 2.4. Storage Conditions

All eggs were stored in a climate-controlled cabinet (HD-960L-3in1; HD Kuluçka Makineleri, Konya, Türkiye) at 9–12 °C and 75% relative humidity, in a pointed-end-up orientation, and turned automatically 24 times per day through 45° arcs. Storage temperature and humidity were verified twice daily. Prior to incubation, all eggs were fumigated (formaldehyde + potassium permanganate, 2:1 *v*/*w*, 10 min).

### 2.5. SPIDES Protocol

In SPIDES subgroups, eggs were transferred from the storage cabinet to a setter incubator (T4800C; Çimuka Kuluçka Makineleri, Ankara, Türkiye) for 2 h at 37.7 °C and 59% relative humidity. SPIDES was applied commencing on day 28 of storage, repeated in 7-day intervals thereafter (days 28, 35, 42, and 49 for the 56-day group; days 28, 35, and 42 for the 49-day group; days 28 and 35 for 42-day group; day 28 for 35-day group). A 30 min equilibration period at 20–25 °C was applied before and after each SPIDES episode to minimize thermal shock.

### 2.6. Incubation and Hatching

After storage, all eggs were transferred to the setter (37.7 °C, 59% RH, turned 6 times/day at 45°) for 21 days. On day 21, eggs were individually weighed (Wt) and transferred to individual mesh bags placed in the hatcher (37.2 °C, 72% RH, no turning). Hatch checks were performed at 09:00 and 21:00 h. Although the normal incubation period of chukar partridge eggs is approximately 24 days [26], incubation was continued until day 26 to account for potential delays associated with prolonged egg storage, at which point unhatched eggs were candled and opened for embryonic mortality classification.

### 2.7. Measurements

At the end of storage period, 20 eggs from each subgroup were selected for egg quality measurements. Measurements included albumen height, albumen length and width (mm), yolk height and yolk diameter (mm) using digital caliper (Kanon EMS-150; Nakamura Mfg. Co., Ltd., Tokyo, Japan). Egg, yolk, albumen, and shell weights were measured to ±0.01 g. The following indices were calculated:

- Haugh unit (HU) = 100 × log(albumen height + 7.57 − 1.7 × egg weight^0.37^);
- Albumen index (%) = albumen height/[(albumen length + albumen width)/2] × 100;
- Yolk index (%) = yolk height/yolk diameter × 100;
- Albumen ratio (%) = albumen weight/egg weight × 100;
- Yolk ratio (%) = yolk weight/egg weight × 100;
- Yolk: albumen ratio (%) = yolk weight/albumen weight × 100;
- Shell ratio (%) = shell weight/egg weight × 100.

Weight losses were calculated from three weighing points:

W_0_ (pre-storage), Ws (post-storage/pre-incubation), and Wt (transfer, day 21 of incubation): storage weight loss (%) = (W_0_ − Ws)/W_0_ × 100; transfer weight loss (%) = (Ws − Wt)/Ws × 100; total weight loss (%) = (W_0_ − Wt)/W_0_ × 100.

Hatching performance parameters included hatchability (%) and hatchability of fertile eggs (%). Embryonic mortality was classified as early (days 0–9), mid (days 9–21), late (day 21 onward), and pipped (pip mortality).

Chick quality was assessed using the Tona scoring system [27], with complete quality records available for 390 of the 399 hatched chicks.

### 2.8. Statistical Analysis

All analyses were performed in Python 3.9.13 using pandas, NumPy, SciPy, and statsmodels libraries. Normality was assessed by the Shapiro–Wilk test and homogeneity of variance by the Levene test. Continuous variables, including chick quality scores, were analyzed using a General Linear Model (GLM) that included storage duration, SPIDES, and their interaction as fixed effects. Multiple comparisons were performed using Bonferroni correction where significant main effects were detected. Categorical data (hatchability, hatchability of fertile eggs, embryonic mortality) were compared by chi-square test. Statistical significance was set at *p* < 0.05. Results are expressed as means ± standard error of the mean (SEM) for continuous variables and as percentages for proportional data.

## 3. Results

### 3.1. Egg Quality Characteristics

The effects of storage duration and SPIDES on albumen index, yolk index, and Haugh unit are presented in Table 1. Storage duration significantly affected Haugh unit, albumen index, and yolk index (*p* < 0.001). The highest Haugh unit (82.69), albumen index (2.38%), and yolk index (38.76%) were observed in eggs stored for 35 days. A significant interaction between storage duration and SPIDES was detected for the yolk index (*p* < 0.01). This interaction was mainly due to the higher yolk index in the SPIDES group at 42 days of storage (*p* < 0.001), whereas no significant interaction was observed for Haugh unit or albumen index.

Effects of storage duration and SPIDES on egg component weights are presented in Table 2. Albumen weight declined significantly at 56 days of storage compared with shorter storage periods (*p* < 0.01), whereas egg weight, yolk weight, and shell weight were not significantly affected by storage duration. SPIDES did not significantly influence any weight parameter, and no significant SL × SPIDES interactions were detected.

Effects of storage duration and SPIDES on egg component ratios are presented in Table 3. Yolk ratio (*p* < 0.05), albumen ratio (*p* < 0.01), and yolk-to-albumen ratio (*p* < 0.01) were significantly affected by storage duration. The highest yolk ratio (38.54%) and yolk-to-albumen ratio (75.45) and the lowest albumen ratio (51.69%) were recorded at 56 days of storage. Shell ratio was not significantly affected by storage duration. SPIDES did not significantly affect any component ratio, and no significant SL × SPIDES interactions were detected.

### 3.2. Weight Losses, Incubation Length, and Chick Weight

Weight loss and incubation parameters are summarized in Table 4. Storage duration significantly affected pre-incubation egg weight (*p* < 0.001), storage weight loss (*p* < 0.001), transfer weight loss (*p* < 0.001), chick weight (*p* < 0.001), and incubation length (*p* < 0.001). Storage weight loss increased progressively from 3.03% at 35 days to 5.06% at 56 days, while transfer weight loss declined from 13.38% to 9.86% over the same interval. Chick weight was lowest in the 49-day group (11.58 g). Incubation length was longest in the 56-day group (25.40 days).

SPIDES significantly affected chick weight and incubation period (*p* < 0.001). Chicks hatched from SPIDES-treated eggs were heavier than those in the control group (13.19 vs. 12.76 g), and their incubation period was slightly shorter (24.33 vs. 24.38 days). Significant storage duration × SPIDES interactions were detected for weight loss during storage, chick weight, and incubation period (*p* < 0.001), as well as for total egg weight loss (*p* < 0.05). Simple-effect comparisons showed that SPIDES reduced EWLS and TEWL at 35 days and increased EWLS at 56 days (*p* < 0.05). SPIDES also increased hatch weight at 35 and 56 days and affected incubation period at 35, 42, and 56 days (*p* < 0.05).

### 3.3. Hatchability and Embryonic Mortality

The effects of storage duration and SPIDES on hatching performance and embryonic mortality are presented in Table 5 and Table 6. Hatchability decreased significantly after 49 and 56 days of storage, whereas hatchability of fertile eggs was significantly lower at 56 days (*p* < 0.05). Early embryonic mortality was highest after 56 days of storage (*p* < 0.05), whereas middle and late embryonic mortality were not significantly affected by storage duration.

The effect of SPIDES differed according to storage duration (Table 5). After 56 days of storage, SPIDES increased hatchability from 31.25% to 63.75% and hatchability of fertile eggs from 65.79% to 83.61%. It also decreased early embryonic mortality from 31.58% to 9.84% (*p* < 0.05). No significant effect of SPIDES was observed after 35 or 42 days of storage.

As a main effect, SPIDES increased hatchability (*p* < 0.05; Table 6). SPIDES did not significantly affect hatchability of fertile eggs and total embryonic mortality rates.

### 3.4. Chick Quality

Chick quality scores are presented in Table 7. Storage duration significantly affected chick quality score (*p* < 0.05). Chicks hatched from eggs stored for 56 days had lower quality scores than those hatched from eggs stored for 35, 42, or 49 days. SPIDES increased the overall chick quality score (97.72 vs. 96.60; *p* < 0.05). A significant storage duration × SPIDES interaction was also detected (*p* < 0.05). The interaction was mainly associated with the 56-day storage period, when chick quality was higher in the SPIDES group than in the control group (*p* < 0.05).

## 4. Discussion

### 4.1. Egg Quality Characteristics

The decrease in egg quality during storage for 35–56 days may be explained by the loss of CO_2_ through the shell pores. The loss of CO_2_ increases albumen pH and weakens the ovomucin–lysozyme complex responsible for maintaining albumen viscosity. Consequently, albumen height, albumen index, and Haugh unit decrease during storage [8,9]. Similar changes have been reported in partridges [14,15], chickens [28], Japanese quails [29], and pheasants [12].

In the present study, albumen index declined from 2.38% at 35 days to 1.85% at 56 days, and Haugh unit from 82.69 to 78.05—changes that, while statistically significant, were modest relative to the declines observed in broiler and quail eggs over substantially shorter storage periods [28,29]. This attenuated deterioration is consistent with the hypothesis that *A. chukar* eggs possess structural or biochemical features—potentially including greater cuticle stability, differential shell conductance, or a more durable vitelline membrane—that slow the progression of storage-induced damage [13,30]. Whether these properties are solely reflective of egg size and surface area-to-volume ratio or whether they involve species-specific molecular adaptations with evolution remains to be determined.

Yolk index decreased from 38.76% after 35 days of storage to 32.83% after 56 days. This decrease may have resulted from the movement of water from the albumen to the yolk as the vitelline membrane weakens during storage [8,31]. At 56 days, albumen ratio decreased, whereas yolk ratio and yolk-to-albumen ratio increased (Table 3). These changes indicate a redistribution of water between the albumen and yolk during prolonged storage [31]. A significant storage duration × SPIDES interaction was detected for the yolk index; however, SPIDES had no significant main effect on this variable.

SPIDES did not significantly affect Haugh unit, albumen index, egg component weights, or egg component ratios. Similar results have been reported in broiler eggs, in which SPIDES had limited effects on albumen quality [32,33]. These findings suggest that SPIDES does not prevent the physical changes occurring within the egg during prolonged storage. Its beneficial effects may therefore be related mainly to embryonic viability rather than the maintenance of internal egg quality.

### 4.2. Hatching Performance and Embryonic Mortality

Hatchability remained relatively high after 35 and 42 days of storage and decreased at 49 and 56 days, whereas hatchability of fertile eggs remained relatively high through 49 days and was lowest at 56 days (Table 6). Previous studies reported that chukar partridge eggs could be stored for up to 28 days without a marked reduction in hatchability [13,16,34]. The present results indicate that these eggs retain their hatching capacity for up to 42 days under the storage conditions used in this study. This period is longer than those commonly reported for chickens [10], game quail [18], and some other partridge species [17]. However, differences among studies in breeder age, egg handling, storage conditions, and incubation procedures should be considered.

The decrease in hatchability after 49 and 56 days of storage was accompanied by an increase in early embryonic mortality, which reached 18.18% after 56 days (Table 6). Prolonged storage may reduce blastoderm viability and impair the resumption of embryonic development after incubation begins [35,36]. Similar increases in early embryonic mortality after prolonged storage have been reported in broiler breeder [10,37] and red-legged partridge eggs [25]. In contrast, middle and late embryonic mortality were not significantly affected by storage duration in the present study. Embryonic malformations were not specifically evaluated in the present study; therefore, potential effects of prolonged storage and SPIDES on developmental abnormalities require further investigation.

The most practically relevant finding regarding SPIDES was its marked effect at 56 days of storage (Table 5), where it increased hatchability from 31.25% to 63.75% and hatchability of fertile eggs from 65.79% to 83.61%, while reducing early embryonic mortality from 31.58% to 9.84% (*p* < 0.05). These results are consistent with the mechanistic explanation proposed by Dymond et al. [21] and Brady et al. [23]: SPIDES may facilitate the transition of the blastoderm from the more vulnerable EGK X stage to the more stress-resistant EGK XII–XIII range, reduces apoptotic signaling, and maintains a greater proportion of viable cells available to reinitiate development upon full incubation. The progressive, interval-based design of the SPIDES protocol applied here—initiated at day 28 and delivered every 7 days for 2 h—appears well-matched to the biology of the chukar partridge, avoiding the overshoot risk associated with single long-term pre-storage incubation (PRESI) identified in red-legged partridge [25].

The absence of significant SPIDES effects at 35 and 42 days supports the interpretation that this intervention is not warranted as a routine procedure for short-to-moderate storage durations, at which embryonic resilience remains adequate. This operational refinement has practical implications: SPIDES represents an additional labor and energy cost, and its application should be reserved for scenarios in which storage duration is expected to exceed 42–49 days.

### 4.3. Weight Loss Dynamics

The increase in storage weight loss from 3.03% at 35 days to 5.06% at 56 days reflects the cumulative effect of sustained shell conductance and declining cuticle integrity over time [38]. Notably, transfer weight loss declined over the same interval (13.38% to 9.86%), such that total weight loss remained statistically unchanged across groups (Table 4). This redistribution of water loss between the storage and incubation phases suggests that long-term storage alters the internal egg environment in ways that modify subsequent evaporative dynamics during incubation—potentially through changes in albumen viscosity, vitelline membrane permeability, or sub-embryonic fluid composition [39]. These findings support a cautious interpretation: the absence of increased total weight loss across storage groups does not imply an absence of embryotoxic hydration imbalance, but rather a temporal redistribution of equivalent total water loss.

The significant reduction in chick weight at 49 days (11.58 g) relative to other groups, together with the extension of incubation length to 25.40 days at 56 days, is consistent with reports that long-term storage retards embryonic development and delays hatching in multiple species [10,27]. The improvement in chick weight and shortening of incubation length in SPIDES-treated eggs suggests that periodic thermal stimulation partially corrects this developmental delay, likely by maintaining a higher proportion of viable blastoderm cells that can initiate timely development upon full incubation.

### 4.4. Chick Quality

The decline in chick quality score at 56 days of storage (Table 7) reflects the cumulative impact of storage-induced embryonic stress on developmental quality at hatch. This outcome aligns with previous reports documenting reduced chick quality scores, impaired navel healing, and reduced vitality in chicks from long-stored eggs [27,40]. The significant improvement in the chick quality score with SPIDES (97.72 vs. 96.60; *p* < 0.05), together with the significant SL × SPIDES interaction, is consistent with the broader framework of SPIDES as a strategy for maintaining embryonic developmental competence rather than preventing physicochemical egg quality deterioration per se.

The storage-duration-dependent nature of the SPIDES benefit—most evident at the longest storage duration—reinforces the threshold model: SPIDES provides measurable value only when storage-induced embryonic stress exceeds the species’ intrinsic compensatory capacity, which in *A. chukar* appears to occur beyond 42–49 days. This finding is in agreement with reports that short thermal treatments during long storage improve chick quality in broiler breeders [21,33,41], while emphasizing that the effect is conditional on storage severity.

## 5. Conclusions

The hatchability of fertile eggs remained relatively high for up to 49 days, although the overall hatchability decreased when storage reached 49 days. The greatest reduction in hatching performance and the highest early embryonic mortality were observed after 56 days of storage. SPIDES improved hatching performance after prolonged storage, with its clearest effect observed after 56 days. It also increased the overall chick quality score but had limited effects on physical egg quality traits. These findings indicate that chukar partridge eggs retain their viability during long storage periods and that SPIDES helps reduce the negative effects when prolonged storage cannot be avoided. Further studies are needed to determine the most suitable SPIDES protocol for chukar partridge and other partridge species.

## Figures and Tables

**Table 1:** Effects of storage duration and SPIDES on albumen index, yolk index, and Haugh unit in chukar partridge eggs.

Storage Length (d)	SPIDES	n	Haugh Unit	Albumen Index (%)	Yolk Index (%)
35	Control	20	82.89	2.39	38.78
	SPIDES	20	82.50	2.37	38.73
42	Control	20	80.35	2.14	33.96 ^b^
	SPIDES	20	82.88	2.09	38.77 ^a^
49	Control	18	81.45	2.27	33.43
	SPIDES	18	79.73	2.19	31.92
56	Control	20	76.80	1.85	33.18
	SPIDES	18	79.43	1.85	32.44
Storage Length (d)					
35		40	82.69 ^a^	2.38 ^a^	38.76 ^a^
42		40	81.61 ^a^	2.12 ^bc^	36.36 ^b^
49		36	80.59 ^ab^	2.23 ^b^	32.67 ^ab^
56		38	78.05 ^b^	1.85 ^c^	32.83 ^c^
SEM			0.37	0.03	0.29
*p*			***	***	***
SPIDES					
	Control	78	80.34	2.16	34.87
	SPIDES	76	81.22	2.13	35.64
SEM			0.26	0.02	0.21
*p*			n.s.	n.s.	n.s.
Interaction					
SL × SPIDES			n.s.	n.s.	**

SPIDES: short periods of incubation during egg storage; ^a, b, c^ values within a column with different superscripts differ significantly (***: *p* < 0.001; **: *p* < 0.01; n.s.: not significant).

**Table 2:** Effects of storage duration and SPIDES on egg component weights (g) in chukar partridge eggs.

Storage Length (d)	SPIDES	n	Egg Weight	Yolk Weight	Albumen Weight	Shell Weight
35	Control	20	20.44	7.45	11.02	1.96
	SPIDES	20	20.10	7.46	10.65	2.00
42	Control	20	20.38	7.67	10.75	1.95
	SPIDES	20	19.86	7.24	10.73	1.89
49	Control	18	19.93	7.29	10.78	1.86
	SPIDES	18	20.59	7.64	11.08	1.88
56	Control	20	19.60	7.43	10.29	1.89
	SPIDES	18	19.26	7.54	9.82	1.91
Storage Length (d)						
35		40	20.27	7.46	10.84 ^a^	1.98
42		40	20.12	7.46	10.74 ^a^	1.92
49		36	20.26	7.46	10.93 ^a^	1.87
56		38	19.44	7.48	10.06 ^b^	1.90
SEM			0.12	0.06	0.09	0.02
*p*			n.s.	n.s.	**	n.s.
SPIDES						
	Control	78	20.09	7.47	10.71	1.92
	SPIDES	76	19.95	7.46	10.57	1.92
SEM			0.09	0.04	0.06	0.01
*p*			n.s.	n.s.	n.s.	n.s.
Interaction						
SL × SPIDES			n.s.	n.s.	n.s.	n.s.

SPIDES: short periods of incubation during egg storage; ^a, b^ values within a column with different superscripts differ significantly (**: *p* < 0.01; n.s.: not significant).

**Table 3:** Effects of storage duration and SPIDES on egg component ratios (%) in chukar partridge eggs.

Storage Length (d)	SPIDES	n	Yolk	Albumen	Shell	Yolk:Albumen
35	Control	20	36.54	53.83	9.63	68.24
	SPIDES	20	37.10	52.97	9.94	70.34
42	Control	20	37.78	52.67	9.56	72.47
	SPIDES	20	36.44	54.07	9.50	67.52
49	Control	18	36.55	54.04	9.41	67.83
	SPIDES	18	37.14	53.68	9.18	69.60
56	Control	20	38.03	52.33	9.64	73.79
	SPIDES	18	39.11	50.98	9.91	77.29
Storage Length (d)						
35		40	36.82 ^b^	53.40 ^a^	9.78	69.29 ^b^
42		40	37.11 ^ab^	53.37 ^ab^	9.53	70.00 ^ab^
49		36	36.85 ^ab^	53.86 ^a^	9.29	68.71 ^b^
56		38	38.54 ^a^	51.69 ^b^	9.77	75.45 ^a^
SEM			0.23	0.23	0.07	0.77
*p*			*	**	n.s.	**
SPIDES						
	Control	78	37.24	53.20	9.56	70.58
	SPIDES	76	37.41	52.95	9.64	71.19
SEM			0.16	0.16	0.05	0.54
*p*			n.s.	n.s.	n.s.	n.s.
Interaction						
SL × SPIDES			n.s.	n.s.	n.s.	n.s.

SPIDES: short periods of incubation during egg storage; ^a, b^ values within a column with different superscripts differ significantly (*: *p* < 0.05; **: *p* < 0.01; n.s.: not significant).

**Table 4:** Effects of storage duration and SPIDES on incubation length and egg weight traits during storage and incubation period of chukar partridge eggs.

Factors		n	EWBS, g	EWBI, g	EWI-21, g	EWLS, %	EWLI-21, %	TEWL, %	HW, g		IL, d
									n	Mean	
SL	35	160	20.94	20.32 ^a^	17.65	3.03 ^c^	13.38	15.90	120	13.29 ^a^	24.00 ^c^
	42	160	20.82	20.06 ^ab^	17.81	3.61 ^b^	11.85	14.93	112	13.31 ^a^	23.98 ^c^
	49	160	20.67	19.84 ^bc^	17.65	4.01 ^b^	11.12	14.66	87	11.58 ^b^	24.39 ^b^
	56	160	20.72	19.64 ^c^	17.71	5.06 ^a^	9.86	14.38	76	13.72 ^a^	25.40 ^a^
	*p*		n.s.	***	n.s.	***	***	n.s.		***	***
	SEM		0.10	0.11	0.13	0.13	0.45	0.49		0.11	0.04
SPIDES	S	320	20.75	19.96	17.73	3.84	11.51	14.85	226	13.19 ^a^	24.33 ^b^
	C	320	20.81	19.97	17.68	4.01	11.60	15.08	169	12.76 ^b^	24.38 ^a^
	*p*		n.s.	n.s.	n.s.	n.s.	n.s.	n.s.		***	***
	SEM		0.07	0.08	0.09	0.09	0.32	0.35		0.09	0.03
SL × SPIDES			n.s.	n.s.	n.s.	***	n.s.	*		***	***

SL: storage length; SPIDES: short periods of incubation during egg storage; S: SPIDES; EWBS: egg weight before storage; EWBI: egg weight before incubation; EWI-21: egg weight at 21 d of incubation; EWLS: egg weight loss during storage; EWLI-21: egg weight loss during first 21 d of incubation; TEWL: total egg weight loss; HW: hatchling weight; IL: incubation length; C: control. ^a, b, c^ Values within a column with different superscripts differ significantly (*: *p* < 0.05; ***: *p* < 0.001; n.s.: not significant).

**Table 5:** The effect of both storage duration and SPIDES on hatchability and embryonic mortality rates in chukar partridge eggs.

Factors				Embryonic Mortality Rates (%)				
SL, d	S	Hatchability, %	HOFE, %	E	M	L	P	T
35	C	68.75	84.62	4.62	3.08	7.69	0.00	15.39 ^a^
	S	81.25	92.86	2.86	0.00	4.29	0.00	7.15 ^b^
42	C	67.50	91.53	3.39	3.39	1.69	0.00	8.47 ^b^
	S	75.00	86.96	2.90	1.45	7.25	1.45	13.05 ^a^
49	C	43.75 ^b^	85.37	2.44	4.88	4.88	2.44	14.64
	S	67.50 ^a^	85.71	7.94	3.17	0.00	3.17	14.28
56	C	31.25 ^b^	65.79 ^b^	31.58 ^a^	2.63	0.00	0.00	34.21 ^a^
	S	63.75 ^a^	83.61 ^a^	9.84 ^b^	0.00	4.92	1.64	16.40 ^b^

SPIDES: short periods of incubation during egg storage; SL: storage length; HOFE: hatchability of fertile eggs; E: early; M: middle; L: late; P: pipped; T: total; S: SPIDES; C: control. ^a, b^: Values within a column with different superscripts differ significantly (*p* < 0.05).

**Table 6:** Main effect of storage duration and SPIDES on hatchability and embryonic mortality rates of chukar partridge eggs.

Factors				Embryonic Mortality Rates (%)				
SL, d	S	Hatchability, %	HOFE, %	E	M	L	P	T
35		75.00 ^a^	88.89 ^a^	3.70 ^a^	1.48	5.93	0.00	11.11 ^a^
42		71.25 ^a^	89.06 ^a^	3.13 ^a^	2.34	4.69	0.78	10.94 ^a^
49		55.63 ^b^	85.58 ^ab^	5.77 ^a^	3.85	1.92	2.88	14.42 ^ab^
56		47.50 ^b^	76.77 ^b^	18.18 ^b^	1.01	3.03	1.01	23.23 ^b^
	S	71.88 ^a^	87.45	5.70	1.14	4.18	1.52	12.54
	C	52.81 ^b^	83.25	8.87	3.45	3.94	0.49	16.75

SPIDES: short periods of incubation during egg storage; SL: storage length; HOFE: hatchability of fertile eggs; E: early; M: middle; L: late; P: pipped; T: total; S: SPIDES; C: control. ^a, b^: Values within a column with different superscripts differ significantly (*p* < 0.05).

**Table 7:** Effects of storage duration and SPIDES on chick quality score in chukar partridge eggs.

Factors		N	Chick Quality (Mean)
Storage Length (d)	SPIDES		
35		118	97.02 ^a^
42		110	98.16 ^a^
49		87	97.33 ^a^
56		75	96.08 ^b^
SEM			0.22
*P*			*
	Control	169	96.60 ^b^
	SPIDES	221	97.72 ^a^
SEM			0.22
*P*			*
Interaction			
Storage Length × SPIDES			*

SPIDES: short periods of incubation during egg storage; ^a, b^ values within a column with different superscripts differ significantly (*p* < 0.05). *: *p* < 0.05.

## Data Availability

The data presented in this study are available on request from the corresponding author.

## Supplementary Materials

The following supporting information can be downloaded at: https://www.mdpi.com/article/10.3390/ani16182819/s1. Supplementary Table S1. Ingredients and calculated chemical composition of the partridge breeder diet.
