# Supplementary material for: Effects of Short Periods of Incubation During Egg Storage (SPIDES) on Egg Quality, Hatching Performance, and Chick Quality in Long-Term-Stored Chukar Partridge Eggs
Source: Animals (Basel). 2026 Sep 8;16(18):2819. doi: 10.3390/ani16182819 (PMC13603933; doi:10.3390/ani16182819)
Supplement: Supplementary file 1 [file animals-16-02819-s001.zip › animals-4546861-supplementary.pdf]

Supplementary Table S1. Ingredients and calculated chemical composition of the partridge breeder diet.

Ingredients	% (dry matter)
Wheat	37.28
Maize	24.90
Boncalite	5.00
Vegetable oil	3.00
Soybean meal (48%)	19.10
Marble powder	6.37
Dicalcium phosphate 18	2.78
L-lysine hydrochloride	0.88
Salt	0.42
Vitamin–mineral premix[a]	0.25
DL-methionine	0.02
**Calculated nutrient concentration**	
ME (kcal/kg)	2800
CP (% KM)	18
Calcium (%)	3.11
Phosphorus (%)	0.61
Lysine (%)	1.50
Methionine + cystine (%)	0.60

[a] Premix provided the following per kg of diet: Vitamin A, 8,800 IU; vitamin D_3, 2,200 IU; vitamin E, 11 mg; nicotinic acid, 44 mg; calcium D-pantothenate, 8.8 mg; riboflavin, 4.4 mg; thiamine, 2.5 mg; vitamin B_{12}, 6.6 mg; folic acid, 1 mg; D-biotin, 0.11 mg; choline, 220 mg; Mn, 80 mg; Cu, 5 mg; Fe, 60 mg; Zn, 60 mg; Co, 0.20 mg; iodine, 1 mg; Se, 0.15 mg.
